# *ADAM33* gene polymorphisms in Southwestern Iranian patients with asthma 

**DOI:** 10.22038/IJBMS.2018.25553.6312

**Published:** 2018-08

**Authors:** Shirin Farjadian, Mozhgan Moghtaderi, Bent-Alhoda Hoseini-Pouya, Azin Ebrahimpour, Mahboubeh Nasiri

**Affiliations:** 1 Department of Immunology, Shiraz University of Medical Sciences, Shiraz, Iran; 2 Allergy Clinic of Ali-Asghar Hospital, Shiraz University of Medical Sciences, Shiraz, Iran

**Keywords:** *ADAM33*, Asthma, rs511898, rs3918396, rs2280089, rs2280091

## Abstract

**Objective(s):**

Asthma, the most frequent chronic respiratory disease, results from a complex interaction between multiple genes and environmental factors. To date, more than 100 candidate genes and single nucleotide polymorphisms (SNPs) have been reported to be associated with asthma. One of the discovered genes related to asthma is *ADAM33*. However, the relationship between *ADAM33* gene polymorphisms and asthma is controversial. The aim of this study was to investigate the association between four *ADAM33* gene SNPs and susceptibility to asthma in patients from southwestern Iran.

**Materials and Methods:**

*ADAM33* gene polymorphisms at positions T+1 (rs2280091), T1 (rs3918396), S1 (rs2280089), and F+1 (rs511898) were examined in 150 patients with asthma and 149 age- and sex-matched healthy controls with a PCR-RFLP method.

**Results:**

There were no differences between patients and controls in allelic or genotype frequencies of *ADAM33* SNPs. We found no associations between allelic or genotype distribution of the SNPs and spirometry indices, concomitant involvement of other allergic diseases, or exposure to cigarette smoke. In contrast to H4 haplotype, which appeared to be protective against asthma, inheritance of H2 and H3 haplotypes increased the risk of asthma up to 2–3 folds.

**Conclusion:**

*ADAM33* gene polymorphisms appear to play a partial role in asthma susceptibility, investigation of expression changes in this gene in response to environmental factors or the local formation of a soluble form of the molecule in the lung can be helpful to elucidate the impact of this molecule in the induction of asthma.

## Introduction

Asthma is the most frequent chronic respiratory disease, affecting more than 300 million people around the world (1). This heterogeneous disease is classified into allergic and nonallergic types, which share common symptoms. Generally, extrinsic irritants such as tobacco smoke, plant pollen, animal fur, dust mite feces, some kinds of food, exhaust fumes, or certain chemicals stimulate allergic asthma in genetically predisposed individuals, whereas nonallergic or intrinsic asthma is usually triggered by infections and physical or emotional stress (2). In contrast to the relatively stable prevalence of nonallergic asthma of around 3.4%–3.8%, the prevalence of allergic asthma increased from 5.0% in 1996 to 6.0% in 2006 and 7.3% in 2016 (1).

Asthma results from a complex interaction between multiple genes and environmental factors. So far more than 100 candidate genes and single nucleotide polymorphisms (SNPs) are reported to be associated with asthma (3). Familial aggregation of asthma with a higher concordance between monozygotic twins (0.74) than dizygotic twins (0.35), indicates the presence of a strong genetic influence in the induction of asthma (4).

In 2002, *ADAM33 *(disintegrin and metallopro-teinase domain-containing protein 33) was reported to be a genetic risk factor for susceptibility to asthma (5). The *ADAM33 *gene comprises 23 exons extending through a 14-kb segment on chromosome 20p13 and encodes a membrane-anchored metalloprotease which may exert its main effect by processing other molecules, e.g., growth factors and cytokines, linked to airway remodeling (6). In addition to the membrane-bound molecule, secreted and intracellular isoforms can be produced as further splice variants. Variants lacking the metalloproteinase domain have also been reported which may mediate other activities of the molecule. *ADAM33* gene polymorphisms may affect the fate of *ADAM33* transcripts through their effects on mRNA splicing, mRNA stability, and the selection of transcripts for export to the cytoplasm (7). *ADAM33* is known to be involved in branching morphogenesis in the fetal lung, and polymorphic variations in this gene are associated with the pathophysiology of chronic obstructive pulmonary disease, airway remodeling, and hyperresponsiveness in asthma (8-10).

Because the strength of association between asthma and different SNPs in the *ADAM33* gene was reported to vary in different populations, the aim of the present study was to investigate the association between four *ADAM33* SNPs and susceptibility to asthma in a sample of patients from southwestern Iran.

## Materials and Methods

***Patients and controls***

The participants were patients who were referred to allergy clinics affiliated with Shiraz University of Medical Sciences (Fars province, southwestern Iran) with a diagnosis of asthma according to Expert Panel Report 3 criteria (11). The study protocol was approved by the Ethics Committee of our university, and 150 patients with mild to moderate persistent asthma were included in the study after written informed consent was obtained from patients or their parents. Patients with any underlying disease except asthma were excluded from the study. Information was recorded about demographic characteristics, cigarette smoke exposure, family history of atopy and concomitant involvement of allergic rhinitis, atopic dermatitis, and urticaria. 

By public announcement, a total of 149 age- and sex-matched (±1 year) unrelated healthy volunteers of the same ethnicity as the patients and with no personal or family history of asthma or other atopic diseases, were recruited as a control group and offered free screening for asthma. Blood samples (1 ml) for genetic analysis were obtained from patients and controls with EDTA as an anticoagulant.

***Pulmonary function tests***

Pulmonary function tests were performed by spirometry (Cosmed, Rome, Italy) on patients older than 6 years. Forced vital capacity (FVC), forced expiratory volume in 1 second (FEV_1_), ratio of forced expiratory volume in 1 second to forced vital capacity (FEV_1_/FVC) and peak expiratory flow (PEF) were measured, and spirometry indices <80% of the predicted values were considered abnormal (12).

***Genotyping of ADAM33 SNPs***

Genomic DNA was extracted from 200 µl of blood with a DNA extraction kit (GeNet Bio, Nonsan, South Korea) and four SNPs in the *ADAM33* gene previously reported to be associated with asthma in some populations (5,13) were analyzed with a polymerase chain reaction-restriction fragment length polymorphism (PCR-RFLP) method (Table 1). 

The polymorphic sequence-containing regions were amplified by PCR in a total volume of 25 μl containing 30 ng genomic DNA, 12.5 μl 2x master mix (Ampliqon, Odense, Denmark) and 0.5 μmol of each primer (Bioneer, Daejeon, South Korea). Cycling conditions were as follows: one cycle at 95 ^°^C for 5 min, 30 cycles at 95 ^°^C for 30 sec, 62 ^°^C for 30 sec, 72 ^°^C for 30 sec, and a final extension at 72 ^°^C for 10 min. PCR products were digested with related restriction enzymes (Thermo Scientific, Waltham, MA, USA) according to the manufacturer’s protocol, and digested products were then resolved on 2.5% agarose gel. 

***Statistical analysis***

Allele, genotype, and haplotype frequencies of *AMAM33* gene SNPs were calculated using Arlequin v. 3.1 software. Linkage disequilibrium (LD) among SNPs in healthy controls and Hardy-Weinberg compliance were analyzed using Haploview v. 4.2 software. Allele and genotype frequencies were compared between patients and controls – globally and after stratification based on age, sex, and both – using the chi-squared test, and odds ratios (OR) with a 95% confidence interval (CI) were calculated using Epi-info v. 7 software. Relationships between allele and genotype frequencies in each SNP and each spirometry parameter (based on a cut-off value of 80%), cigarette smoke exposure, family history of atopy, or concurrent involvement of other allergic diseases were also analyzed with the chi-squared test. Values of *P*<0.05 were considered statistically significant. 

## Results

The demographic and clinical characteristics along with the spirometry results in patients with asthma are shown in Table 2. Allele, genotype, and three-loci haplotype frequencies of the *ADAM33 *gene SNPs in patients and healthy controls are summarized in Table 3. There were no differences between patients and controls in allelic or genotype frequencies of the SNPs, even after data stratification based on age, sex, and both. We found no associations between allelic or genotype distribution of the SNPs and spirometry indices, family history of atopy, concomitant involvement of other allergic diseases, or exposure to cigarette smoke. 

All SNPs met the criteria for the Hardy-Weinberg equilibrium except for rs511898 (Table 4). This SNP was omitted from subsequent linkage and haplotype analyses (14). 

**Figure 1:**
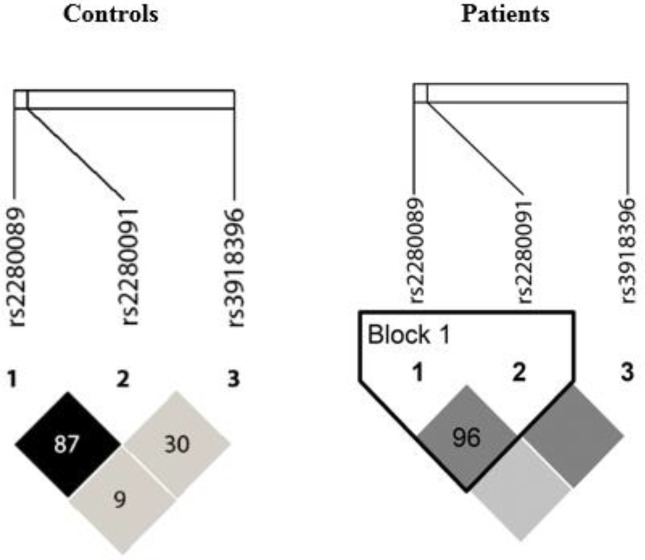
Linkage disequilibrium analysis of *ADAM33* gene SNPs in patients with asthma and healthy controls from southwestern Iran

The results of linkage analysis for the three remaining SNPs showed a linkage between rs2280089 and rs2280091 (Figure 1). The results of haplotype analysis revealed that H5 (0.68%) was observed exclusively in patients, whereas H6 (4.8%), H7 (2.5%), and H8 (1.9%) were seen only in healthy controls. The frequency of H2 and H3 haplotypes were significantly higher in patients than controls (25.65% vs. 14.75%, *P*=0.00046 and 16.65% vs. 7.11%, *P*=0.00013, respectively), and inheritance of these haplotypes was associated with higher risk of asthma up to 2–3 folds. Haplotype H4 was more frequent among controls than patients (8.1% vs. 1.68%, *P*=0.0001) and inheritance of this haplotype was associated with decreased risk of asthma about one fifth in patients (Table 3).

## Discussion

Asthma is a chronic inflammatory disorder characterized by obstruction of the bronchial tubes and airway remodeling with increased smooth muscle mass, fibroblast activation, neovascularization, and epithelial alterations. *ADAM33* is preferentially expressed in airway fibroblasts and smooth muscle cells in patients with asthma. 

**Table 1:** ADAM33 gene SNP genotyping by PCR-RFLP: primers, restriction enzymes, and length of the digested product fragments

**ADAM33 SNP**	**Primer sequences**	**Restriction ** **enzyme**	**Recognition site**	**Fragment size (bp)**
T+1 (rs2280089) (intron 20)	F: AGGGTCTGGGAGAAATGGTG R: TCTTTGGAAGCTGAGCGATG	MboII	…GAAG**A**(N)_8_↓… …CTTCT(N)_7_↑…	GG: 424+132 bp AG: 424+132+123+301 bp AA: 301+132+123 bp
T1 (rs2280091) (exon 20)	F: GTGAATATGGTCAGCAGGAG R: GTGACTTGGAGCAGATGG	NcoI	…C↓C**A**TGG… …GGTAC↑C…	GG: 375 bp GA: 375+187+188 bp AA: 187+188 bp
S1 (rs3918396) (exon 19)	F: GTGGCAGCATGGACAGT R: CAGGAGTAGGCTCAGGAAG	BtsCI	…GG**A**TGNN↓… …CCTAC↑NN…	GG: 304 bp GA: 304+153+151 bp AA: 151+153 bp
F+1 (rs511898) (intron 6)	F: AAATACGACTCGAGGC R: GGACTTCTCAACCCACGAG	BsmBI	…**C**GTCTC(N)_1_↓… …GCAGAG(N)_5_↑…	TT: 220 bp TC: 220+183+37 bp CC: 183+37 bp

**Table 2:** Demographic and clinical characteristics of patients with asthma

Age (year)	<13 (n=44)		≥13 (n=106)		
Sex	Female (n=22)	Male (n=22)	Female (n=68)	Male (n=38)
Mean age±SD (years) (Age range)	8.95±2.44 (5 to 12)	8.27±2.34 (5 to 12)	44.17±16.52 (13 to 82)	39.01±19.39 (13 to 82)
Family history	9	12	29	23
Cigarette smoke exposure	19	14	38	11
Concurrent allergic diseases						
Allergic rhinitis	12	12	33	29
Eczema	18	14	9	10
Urticaria	16	12	16	9
Spirometry parameters						
FEV_1_ <80%	4	6	34	22
FVC <80%	4	9	35	20
FEV_1_/FVC <80%	0	0	3	4
PEF <80%	8	12	45	24

**Table 3:** Allele, genotype, and haplotype frequencies of *ADAM33* gene SNPs in Southwestern Iranian patients with asthma and healthy controls

**ADAM33**	**Patients** **(n=150)**	**Controls** **(n=149)**	***P*** **-value**	**OR (CI95%)**
**Alleles**				
**T+1**	**N (F%)**	**N (F%)**		
G	221 (73.6%)	234 (78.5%)	0.19	0.7 (0.5 - 1.1)
A	79 (26.4%)	64 (21.5%)		
**T1**				
G	82 (27.3%)	90 (30.2%)	0.49	0.8 (0.5 - 1.2)
A	218 (72.7%)	208 (69.8%)		
**S1**				
G	248 (82.6%)	255 (85.6%)	0.39	0.8 (0.6 - 1.2)
A	52 (17.4%)	43 (14.4%)		
**F+1**				
T	176 (58.6%)	172 (57.7%)	0.87	1.03 (0.75 - 1.4)
C	124 (41.4%)	126 (42.3%)		
**Genotypes**				
**T+1**				
GG	77 (51.3%)	92 (61.7%)	0.1	0.6 (0.41 - 1.03)
GA	67 (44.7%)	50 (33.6%)		1.5 (1 - 2.5)
AA	6 (4%)	7 (4.7%)		0.8 (0.2 - 2.5)
**T1**				
GG	6 (4%)	8 (5.4%)	0.69	0.7 (0.24 - 2.17)
GA	70 (46.7%)	74 (49.7%)		0.8 (0.56 - 1.39)
AA	74 (49.3%)	67 (44.9%)		1.1 (0.7 - 1.8)
**S1**				
GG	99 (66%)	106 (71.1%)	0.41	0.7 (0.48 - 1.2)
GA	50 (33.3%)	43 (28.9%)		1.2 (0.77 - 2.06)
AA	1 (0.6%)	---		---
**F+1**				
TT	28 (18.6%)	27 (18.1%)	0.7	1 (0.57 - 1.86)
TC	120 (80%)	118 (79.2%)		1 (0.58 - 1.84)
CC	2 (1.3%)	4 (2.7%)		0.4 (0.08 - 2.7)
**Haplotypes T+1/T1/S1**				
**H1:** G A G	166 (55.33%)	181 (60.79%)	0.09	0.8008 (0.5784-1.1087)
**H2:** A G G	77 (25.65%)	44 (14.75%)	0.00046	1.9933 (1.3205-3.0088)
**H3: ** G A A	50 (16.65%)	21 (7.11%)	0.00013	2.6381 (1.5411-4.5161)
**H4:** G G G	5 (1.68%)	24 (8.12%)	0.0001	0.1935 (0.0728-0.5143)
**H5:** A A A	2 (0.68%)	---	0.25	---
**H6:** A G A	---	14 (4.82%)	0.000054	---
**H7:** G G A	---	8 (2.50%)	0.0037	---
**H8:** A A G	---	6 (1.90%)	0.015	---

**Table 4:** Deviation from Hardy-Weinberg equilibrium in the *ADAM33 *gene in patients from southwestern Iran with asthma and healthy controls

**ADAm33** **SNPs**	**Position**	**Alleles**	**HWE**					
			**Patients**			**Controls**		
			Obs HET	Pred HET	*P*-value	Obs HET	Pred HET	*P*-value
**T+1**	3669480	G:A	0.447	0.388	0.1009	0.336	0.337	1.0000
**T1 **	3669587	A:G	0.467	0.397	0.0509	0.497	0.422	0.0474
**S1 **	3671118	G:A	0.333	0.287	0.0713	0.289	0.247	0.0574
**F+1 **	3674438	T:C	0.8	0.485	<0.00001	0.792	0.488	<0.00001

Obs HET: observed heterozygosity; Pred HET: predicted heterozygosity

Although there are data showing a link between *ADAM33* and asthma, the exact role of this gene in the pathophysiology of asthma is not entirely clear (5,6,15). The contribution of *ADAM33* gene polymorphisms to the risk of asthma is controversial. Increased risk of asthma in persons with one or more *ADAM33 *SNP allele(s) has been reported in African American, US white, US Hispanic, Dutch white (16), Icelandic, UK (17), and Japanese populations (18).

We found no correlation between any of the four *ADAM33* SNPs (T1, T+1, S1, and F+1) and asthma in a sample of patients from the population of southwestern Iran. The lack of association between *ADAM33* polymorphisms and asthma was also reported in Australian (19), Chinese Li (20), Korean (21), Indian (22), and Turkish populations (23). In the only related report from Iran we are aware of, associations were found between the rs2280091 C allele and severe asthma, and between the rs2787094 G allele and moderate asthma in a population sample from northeastern Iran (24).

Our results suggest that *ADAM33* gene polymorphisms play a restricted role in susceptibility to asthma. Although there is a possible linkage between undefined causal genes on the short arm of chromosome 20 and certain *ADAM33* SNP alleles or haplotypes, increased levels of *ADAM33* gene expression in response to environmental factors such as cigarette smoke or air pollutants might play a critical role in the induction of asthma. Moreover, the local formation of soluble ADAM33 in airways causes remodeling in the developing lungs, which through interaction with Th2-mediated inflammation makes patients more responsive to low concentrations of allergens (25). In this connection, there is evidence that in-utero and early-life tobacco smoke exposure influences the induction of TGF-β2, which in turn results in ADAM33 shedding and reduced lung function (26). However, in contrast to Reijmerink *et al*, who suggested a role for the interaction of cigarette smoke with certain ADAM33 polymorphisms in the induction of asthma (26), we found no association between the SNPs we studied and exposure to cigarette smoke in our patients with childhood or adulthood asthma. 

In contrast to Jongepier *et al*., who found an association between the S2 minor allele and decreased FEV_1_ (27), and in agreement with El-Falaki *et al* (28), we found no association between any of the four SNPs analyzed here and spirometry parameters. Previously, one study reported an inverse correlation between ADAM33 protein levels in bronchoalveolar lavage fluids and FEV_1_ in patients with asthma (29). However, we are not aware of any reports of a functional correlation between any *ADAM33* SNP alleles and ADAM33 protein levels in bronchial smooth muscle cells, bronchial lavage fluids, or serum in patients with asthma. Unlike Zhang *et al*., who reported associations between some *ADAM33* polymorphisms and concomitant allergic rhinitis and asthma in the Chinese Han population (30), and Matsusue *et al*, who found a link between rs2853209 and atopic dermatitis in Japanese children (31), we found no association between *ADAM33* SNPs and any spirometry indices. However, a potential limitation of our study is that we were unable to collect or process bronchoalveolar lavage fluid from our patients to analyze ADAM33 protein levels.

Like another study, we also found a linkage between rs2280089 and rs2280091 loci (32). As shown in Figure 1, this linkage was stronger in patients (*r*^2^ = 0.96) than controls (*r*^2^ = 0.87).

Although we found no link between asthma and any of the *ADAM33* SNPs analyzed here, GGG haplotype was positively associated with the disease in our patients. This confirms the superiority of haplotype analysis over single-SNP analysis in studies that aim to elucidate the associations among different clinical, genetic, and environmental factors in complex genetic disorders such as asthma (33).

## Conclusion

We found no link between asthma and T+1, T1, S1, or F+1 SNPs in the *ADAM33* gene, however, AGG and GAA haplotypes of the first three SNPs were positively associated with the disease in our patients. *ADAM33* gene SNPs appear to play a partial role in asthma susceptibility, and investigation of expression changes in this gene in response to environmental factors or the local formation of soluble form of the molecule in the lung can be helpful to elucidate the impact of this molecule in the induction of asthma.
